# Equitable bivalent booster allocation strategies against emerging SARS-CoV-2 variants in US cities with large Hispanic communities: The case of El Paso County, Texas

**DOI:** 10.1016/j.idm.2023.07.009

**Published:** 2023-07-20

**Authors:** Francis Owusu-Dampare, Anass Bouchnita

**Affiliations:** Department of Mathematical Sciences, The University of Texas at El Paso, El Paso, 79968, Texas, United States

**Keywords:** Mathematical modeling, COVID-19, Hispanic health disparities, Bivalent boosters

## Abstract

COVID-19 is a disease that disproportionately impacts the Hispanic population, due to the prevalence of certain risk factors and the high number of essential workers in this community. In this work, we analyze the vaccination strategies that would minimize the COVID-19 health disparities in El Paso County, TX, in the context of the emergence of a new highly transmissible and immune-escaping SARS-CoV-2 variant. We stratify an age-structure stochastic SEIR model that tracks the evolution of immunity derived from infections and vaccination according to Hispanic vs non-Hispanic ethnicity and parameterize it to the demographic, health and immunization data of El Paso County, TX. After fitting the model, the results show that increasing vaccination with bivalent boosters by five-fold in anticipation of highly transmissible and immune escaping variants would decrease the cumulative hospital admissions and mortality from Mar 1, 2023, to Dec 31, 2023, by 62.72% and 61.41%, respectively. Further, our projections reveal that the disproportionate impact on the Hispanic community would be eliminated if approximately half of the doses that are given to the non-Hispanic group according to the equal distribution, would be re-allocated to the Hispanic population. Our findings can guide public health officials in US cities with large Hispanic communities and help them design vaccination strategies that minimize COVID-19 health disparities caused by emerging variants using specific vaccination strategies.

## Highlights

• Epidemiological framework parameterized to El Paso County, TX data.
• Model stratification according to Hispanic vs non-Hispanic ethnicity.
• Bivalent booster campaign impact on the emergence of transmissible variants.
• Prioritization of Hispanics in booster allocation eliminates disparities.

## 1 Introduction

The COVID-19 pandemic has had a disproportionate impact on minority populations, particularly the Hispanic/Latinx community. The Hispanic population is the second most impacted ethnic group by the COVID-19 pandemic after the Black community (CDC, 2020a). Several studies have shown that Hispanic populations have experienced higher rates of COVID-19 infection, hospitalization, and mortality compared to non-Hispanic White populations (Rodriguez-Diaz et al., 2020; Chen & Krieger, 2021; Rader et al., 2020). In the United States, this disparity is particularly evident in cities with a large Hispanic population such as El Paso, Texas.

There are several factors contributing to the disproportionate impact of COVID-19 on the Hispanic population. One of the major factors is a higher prevalence of underlying health conditions such as diabetes, obesity, hypertension, and cardiovascular diseases, which increase the risk of severe COVID-19 illness and hospitalization (Maddaloni & Buzzetti, 2020; Chu et al., 2021). Furthermore, the Hispanic population is overrepresented among frontline workers, including essential and service workers, who are at a higher risk of exposure to the virus (Kochhar, 2005). In addition, the limited access to healthcare and health insurance, language barriers, and low health literacy levels make it difficult for Hispanic individuals to receive timely and adequate medical care, resulting in delays in testing, diagnosis, and treatment (Garcia et al., 2017).

Further, vaccine hesitancy, concerns about vaccine safety and efficacy, and misinformation regarding COVID-19 vaccines have contributed to lower vaccination rates among the Hispanic population (Hamel et al., 2021). Finally, living in crowded households or densely populated areas, and limited access to affordable housing and transportation, increase the risk of transmission and exposure to the virus (Maroko et al., 2020).

El Paso, TX is a city located on the border between the US and Mexico. Individuals who identify themselves as Hispanic represent 82.9% of El Paso county's population. The relative hesitancy of Hispanic individuals towards vaccination is not as high in El Paso County as in other areas of Texas. As of March 2023, 98% of the population has received a single dose, while 79.2% are fully vaccinated (DOT, 2023). However, the coverage of boosters has been stalling at 39.6% and only 48% of individuals over 50 have received a second dose (DOT, 2023; IISInfo, 2021). To prevent the spread of Omicron variants, the Food and Drug Administration (FDA) has authorized the use of bivalent boosters. Recent clinical studies have shown that these boosters provide better protection against the circulating Omicron variants than their monovalent counterparts (Kawasuji et al., 2022.). These new boosters are expected to play a central role in mitigating new surges caused by emerging variants.

Mathematical modeling has played a key role in the understanding, forecasting, and mitigation of the COVID-19 epidemic. However, there is a lack of modeling studies that investigate the impact of COVID-19 on specific ethnic, racial or socio-demographic groups. A study that uses multiple models of mixing has concluded that 81% of Hispanics were infected in New York State before the herd immunity threshold was reached, while only 34% non-Hispanics were infected at the same moment (Ma et al., 2021). Using data analysis, another study has revealed that areas, where there is a high risk of testing positive, were marked by a higher proportion of Black individuals (DiMaggio et al., 2020). While an SEIR model has revealed that prioritizing the immunization of essential workers reduced the overall disease burden (Ferranna et al., 2021). The impact of COVID-19 on construction workers was explored in another modeling work (Pasco et al., 2020). It concluded that construction workers had a nearly 5-fold increased risk of hospitalization in central Texas compared with other occupational categories. In another study, the analysis of exposure density highlighted the significant disparities in health outcomes for racial and ethnic minorities and lower-income households (Hong et al., 2021).

This study aims to determine vaccination strategies that minimize the disproportionate burden of new COVID-19 variants on the Hispanic population in El Paso County, TX. We begin by adapting a previously developed epidemiological modeling framework that captures the changes in population immunity derived from infections and vaccinations to the recent genomic and immunological landscape of COVID-19. The framework was previously used to make accurate and time-sensitive projections for the impact of the Omicron subvariants in the US (Bi et al., 2022; Bouchnita et al., 2022a) and in Austin, TX (Bouchnita et al., 2022b). We update the model by considering the impact of the variants BQ.1, BQ.1.1, and XBB.1.5 as well as the effect of bivalent boosters. Further, we stratify it according to the Hispanic and non-Hispanic ethnicity status of individuals. We consider that the Hispanic population has higher hospitalization and mortality rates, as well as a high percentage of essential workers, captured through specific contact matrices. We analyze the relative impact of the emergence of a highly transmissible and escaping variant on the Hispanic population. Then, we consider vaccination scenarios corresponding to campaigns where bivalent booster doses are allocated either equally or with prioritization of the Hispanic population. We determine the most equitable vaccination strategy by comparing the relative COVID-19 burden in the Hispanic vs non-Hispanic groups.

## 2 An age- and ethnicity-structured immuno-epidemiological modeling framework that captures the interplay between circulating variants and population-immunity

### 2.1 Model structure

**Fig. 1:**
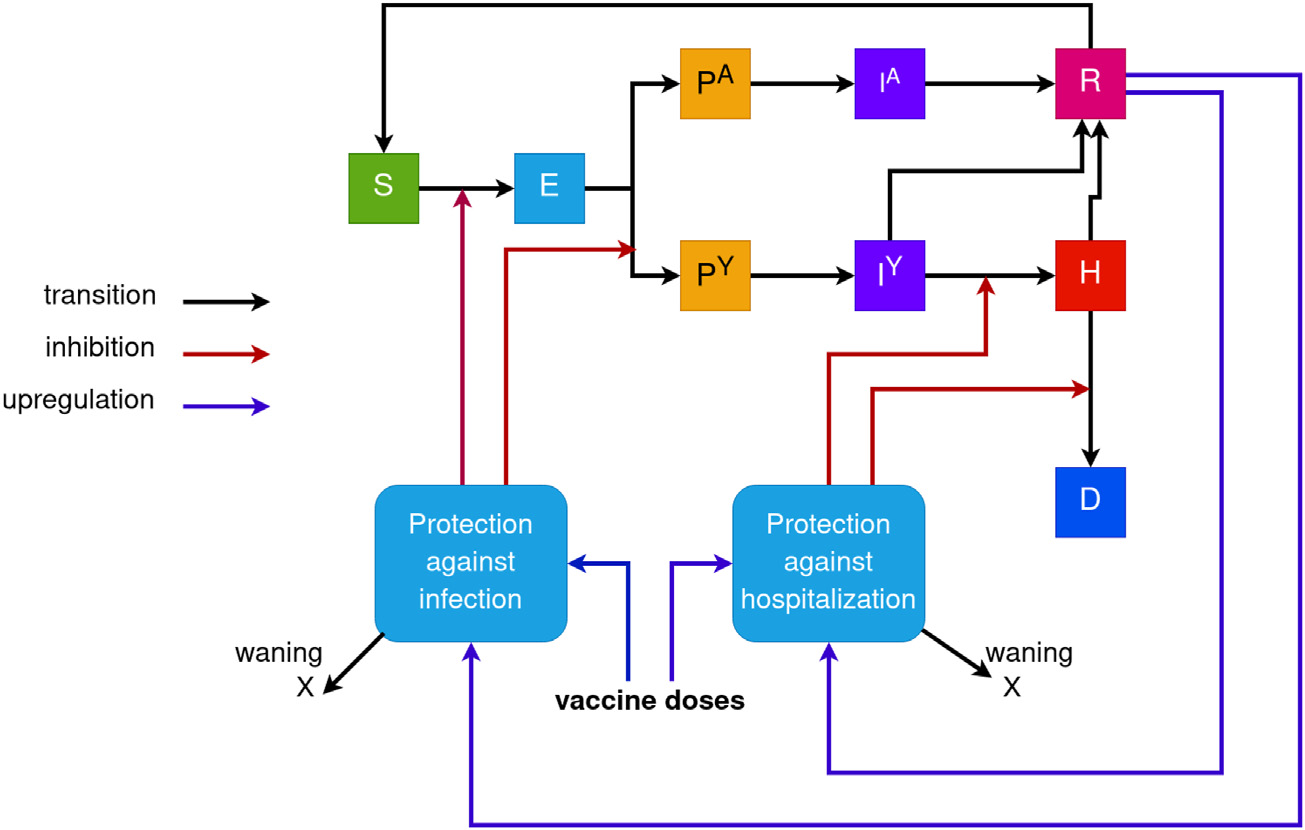
**A scheme representing our COVID-19 transmission model, which explicitly tracks population immunity derived from infections and vaccinations**. When they contract the infection, susceptible people (S) transition to the exposed condition (E). Those who have been exposed move into one of two compartments: pre-symptomatic (PY) or pre-asymptomatic (PA). Pre-asymptomatic cases first pass through the infectious asymptomatic compartment (IA), while those who are pre-symptomatic go to the symptomatic compartment (IY); a portion of these individuals move straight to the recovered compartment, with the remainder moving to the hospitalized compartment (H). Patients in hospitals will either pass away (D) or transfer to the recovered compartment (R). Before returning to the vulnerable compartment, recovered individuals have a brief period of full immunity (S). The model uses two state variables to track the ensuing population-level average protection against infection and against severe disease for each form of immunological exposure (i.e., infection with a specific variant or receipt of a certain type of vaccine). As people recover from diseases and receive vaccinations, these variables rise; they fall according to waning speeds unique to each immune type.

We adapt a previously developed immuno-epidemiological modeling framework (Bi et al., 2022; Bouchnita et al., 2022a) to the recent landscape of COVID-19. Our model describes the changes in the numbers of susceptible individuals, exposed, infectious, hospitalized, deceased and recovered (Fig. 1). In the current implementation of the model, we track the population-immunity levels generated from infections with four variant groups: BA.4/BA.5, BQ.1.1/BQ.1, XBB.1.5, and a hypothetical variant named X, as well as vaccination with both monovalent and bivalent boosters. The average susceptibility and severity of the population depend on the changes in population immunity, which, in turn, depend on the specific variants that are circulating. In all simulations, we consider that cases which are infectious with a new variant called X are imported during four weeks starting from Mar 15, 2023, with an importation rate equal to 5 cases per day. This new variant is assumed to be 55% more transmissible than XBB.1.5 and has a 42.5% chance to escape prior immunity. The model equations, parameter values, and demographic characteristics are provided in the Supplementary material.

### 2.2 Modeling assumptions

Our study is based on the following assumptions regarding the behavior, immunity and variants characteristics:

● The model is initialized by considering the aggregated population immunity levels as of Dec 1, 2022, calculated using a toy immunity model. We fit the transmission and mortality rates using reported hospitalization and mortality data from Dec 1, 2022 to Feb 1, 2023. Then, we assume that the mitigation policies and behavior remain constant and project the number of cases, hospital admissions and deaths from Feb 1, 2023, to Dec 31, 2023.
● As a baseline, we assume that 25% of all infections are reported as cases, though reporting rates can fluctuate according to variant severity.
● We consider that immunity against hospitalization wanes at a slower rate than immunity against infection. For natural immunity, we assume a half-life time of eight months for the waning of immunity against infection, while we consider that protection against hospitalization does not wane. For booster vaccination-derived immunity, we assume a three months half-life time for protection against infection and an eight months half-life time for immunity against hospitalization (Ferdinands et al., 2022; Khoury et al., 2021)
● We no longer consider immunity from vaccination with solely primary series without boosters. That is because the level of this immunity is very low due to the low rate of primary vaccination and the reduced protection offered by primary series against the new immune escaping variants. Hence, we expect the effect of primary vaccination to no longer affect the current epidemiological dynamics.
● As of Dec 1, 2022, we estimated an initial level of immunity against BA.4/BA.5 of 30.4% and against BQ.1/BQ.1.1 of 1.7%. These estimates were made by fitting the previous implementation of the model against hospitalization (Bi et al., 2022; Bouchnita et al., 2022a) and taking into consideration the immune evasiveness of the new variants.
● We assume that age groups interact with one another according to contact rates estimated from the POLYMOD study (Prem et al., 2021). The considered contact matrices are provided in the appendix.
● To simplify the analysis and due to the lack of data, we assume that the variants BA.4 and BA.5 have the same characteristics and impact, and treat them as a single variant. We also treat BQ.1 and BQ.1.1 as a single variant.

### 2.3 Variant growth and characteristics

**Fig. 2:**
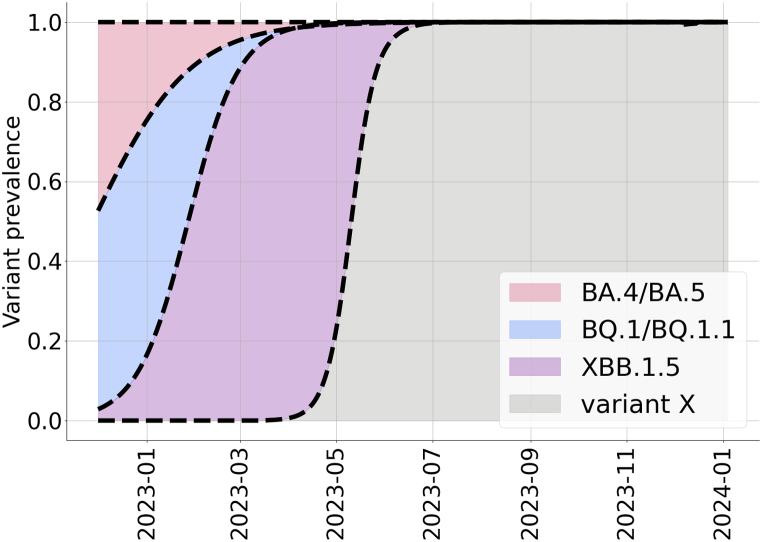
**Estimated ascent of the Omicron subvariants BA**.**4/BA**.**5, BQ**.**1/BQ**.**1**.**1, XBB**.**1**.**5, and the hypothetical variant X in El Paso County, TX**. Values represent the proportion of cases caused by the Omicron subvariants or variant X. Logistic curves for the variants BA.4/BA5, BQ.1/BQ.1.1, and XBB.1.5 were fitted to the measured specimens, according to Nowcast the dashed line is the fitted logistic curve (CDC, 2020b). The growth function of variant X was simulated using a separate two-strain model.

The model describes the impact of four groups of variants which are considered to be circulating as of December 2022: BA.4/BA.5, BQ.1/BQ.1.1, XBB.1.5, and a hypothetical variant X. We assume that the prevalence of each Omicron subvariant is described using a logistic curve, fitted to genomic surveillance data reflecting the relative frequency of Omicron among SARS-CoV-2 specimens sequenced, available through the CDC's COVID Data Tracker (CDC, 2020b). Then, we model the prevalence of variant X using a simplified two-strain model parameterized to the infection cases and immunity levels at El Paso County, TX. We assume that variant X has a 55% transmissibility advantage and 42.5% immune escape against immunity derived from infection with previous subvariants. The considered proportions of infections that are considered for each variant are shown in Fig. 2. We summarize the characteristics of each of the considered variants with respect to transmissibility and immune escape in Table 1.

**Table 1:** The assumed characteristics of the considered variants in the model with respect to transmissibility and immune escape advantages.

	Transmissibility	Immune escape	Reference
BA.5/BA.5	0%	40%	Cao et al. (2022)
BQ.1/BQ.1.1	0%	30%	(Uraki et al., 2023; Zhu et al., 2023)
XBB.1.5	0%	30%	(Uraki et al., 2023; Zhu et al., 2023)
Variant X	55%	42.5%	Assumed

### 2.4 Vaccination scenarios

The objective of this study is to analyze the equity of vaccination strategies that aim to mitigate the emergence of variant X. We consider three vaccination strategies corresponding to vaccination campaigns that aim to increase the daily uptake of bivalent boosters by five-fold, starting from Mar 1, 2023. This policy is continued until 55% of individuals from 5 to 49 and 75% of individuals over 50 receive one bivalent booster. To determine the most equitable strategy, we consider different allocation modes of booster doses among Hispanic and non-Hispanic individuals. Under the first vaccination scenario, we assume that doses are distributed among individuals regardless of their Hispanic status. For the second vaccination scenario, we consider that one-fourth of the doses that should be assigned to non-Hispanic individuals in the case of equal distribution will be allocated to Hispanic individuals. For the third vaccination scenario, we consider that half of the doses that are initially assigned to the non-Hispanic individuals will be redistributed among the more vulnerable Hispanic group. The vaccination scenarios were designed in order to estimate to which extent increasing the uptake of bivalent booster doses by the Hispanic population would minimize COVID-19 Hispanic disparities in the advent of the emergence of a new variant. To estimate the effectiveness of the three vaccination strategies in mitigating the emergence of variant X, we consider one additional scenario where the uptake of bivalent boosters is maintained until reaching coverage levels similar to those reached by the monovalent booster campaign. A summary of the considered vaccination scenarios is provided in Table 2.

**Table 2:** The considered vaccination scenarios which correspond to campaigns that promote the uptake of bivalent among Hispanic and non-Hispanic individuals. For scenarios 1, 2, and 3, the vaccination program stops when 55% of the individuals from 5 to 49 and 75% of individuals over 50 receive a bivalent booster.

	Policy after Mar 1, 2023	Allocation mode among Hispanic vs non-Hispanic individuals
Baseline scenario	Same uptake	Equal distribution
Scenario 1	Five-fold increase	Equal distribution
Scenario 2	Five-fold increase	25% of the allocated doses to the non-Hispanic group are given to Hispanic individuals
Scenario 3	Five-fold increase	50% of the allocated doses to the non-Hispanic group are given to Hispanic individuals

### 2.5 Differences in disease transmission and severity between the Hispanic and non-Hispanic groups

El Paso, TX is a city with a majority of individuals identifying themselves as Hispanic/Latinx. Available data suggest that vaccine hesitancy among Hispanic individuals is not as high as in other places. Hence, we assume the same level of immunization among the Hispanic and non-Hispanic communities at the beginning of each simulation. We consider that Hispanic individuals have a 1.8-fold and 1.7-fold increase in the rates of hospitalization and death than non-Hispanic persons, respectively (CDC, 2023) (NCHS/DVS, 2020). Next, we describe the effect of the increased relative number of essential workers in the Hispanic population through modified contact matrices. It was shown that essential workers make 4.5 more contacts than non-essential workers per day (Thomas et al., 2021.). Further, 59.25% of Hispanic workers are essential while 49.67% of non-Hispanic employers are frontline workers. To reflect this effect, we modify the used contact matrix for the Hispanic population, such that every Hispanic individual makes a higher number of contacts at work.

## 3 Results

### 3.1 Impact of the vaccination campaign on the burden cause by variant X in El Paso county, TX

**Fig. 3:**
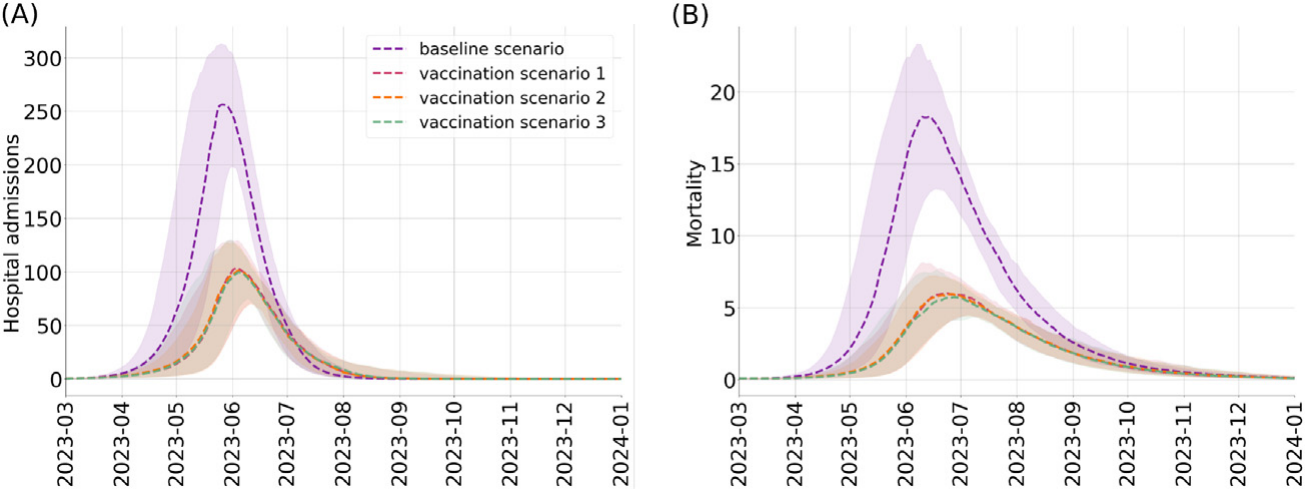
Projected COVID-19 new hospitalizations (left) and deaths (right) in the US under four booster uptake scenarios (Table 2). We compare the impact of vaccination campaigns with bivalent boosters that continue with the same rollout (purple) or increase the rollout and distribute it in different ways among Hispanic and non-Hispanic individuals (pink, orange and green). Ribbons indicate 95% confidence intervals.

The model was fitted using hospitalizations and mortality data from Dec 01, 2022, to Feb 1, 2023. Then, we projected the number of hospital admissions and deaths by assuming the same transmission, hospitalization, and mortality rate from Feb 2, 2023, to Dec 31, 2023. Under the considered scenarios, we run 100 stochastic simulations and compute the 5%, 50%, and 95% quantiles. In the absence of variant X emergence, we project that the number of cases and hospital admissions will continue to go down for the next few months. Fig. 3 shows the 7-day rolling averages of the projected numbers of hospital admissions and deaths from Mar 1, 2023, to Dec 31, 2023. Our projections suggest that in the absence of any vaccination campaign, the emergence of variant X would cause a surge of hospital admissions that will peak on May 23, 2023. At this date, hospital admissions will reach 253 (95% CrI: 198–313). Incident deaths are expected to reach their peak numbers a few weeks later on Jun 11, 2023. The reported peak mortality numbers will be 18 (95% CrI: 13–23). The vaccination campaign will delay the peak and reduce its magnitude. Under the vaccination scenario, the peak hospital admissions will reach 103 (95% CrI: 71–121) on Jun 4, 2023. The incident deaths will reach maximal values of 5 (95% CrI: 4–8) on Jun 17, 2023.

Table 3 presents the cumulative number of hospital admissions and deaths due to COVID-19 for four different vaccination scenarios. The baseline scenario shows that the expected number of hospital admissions and deaths in the absence of any vaccination efforts will be 10895 (95% CrI: 6333 - 17,273) and 1294 (95% CrI: 856 - 1842). The table suggests that increasing vaccine coverage can substantially reduce the number of hospital admissions and deaths due to COVID-19 by approximately 62.72% and 61.41%. Prioritizing Hispanic individuals in the administration of doses will not have a significant impact on the overall number of hospital admissions and deaths. That is because the number of daily doses administered to non-Hispanics is not sufficiently high. Further, the fast immunization of Hispanic individuals using vaccination with bivalent boosters will slow down the development of immunity derived from infections with variant X. Still, Scenario 3, which assumes that half of the doses that should be given to the non-Hispanic subpopulation under the equal distribution assumption to be allocated to the Hispanic individuals, shows the highest reduction in hospital admissions and deaths.

**Table 3:** Projected cumulative COVID-19-associated hospital admissions and deaths between Mar 1, 2023, and Dec 31, 2023, in El Paso County, TX under four vaccination scenarios. The assumptions considered in the scenarios are provided in Table 1. Values are medians and 95% prediction intervals based on 100 stochastic simulations.

Vaccination scenario	Cumulative admissions	Cumulative deaths
Baseline scenario	10,895 (95% CrI: 6333 - 17,273)	1294 (95% CrI: 856 - 1842)
Scenario 1	4895 (95% CrI: 2361 - 8242)	519 (95% CrI: 330–784)
Scenario 2	4922 (95% CrI: 2510 - 7770)	527 (95% CrI: 323–731)
Scenario 3	4815 (95% CrI: 2418 - 8422)	503 (95% CrI: 320–774)

### 3.2 Equity analysis of bivalent booster vaccination strategies against variant X emergence in El Paso county, TX

**Fig. 4:**
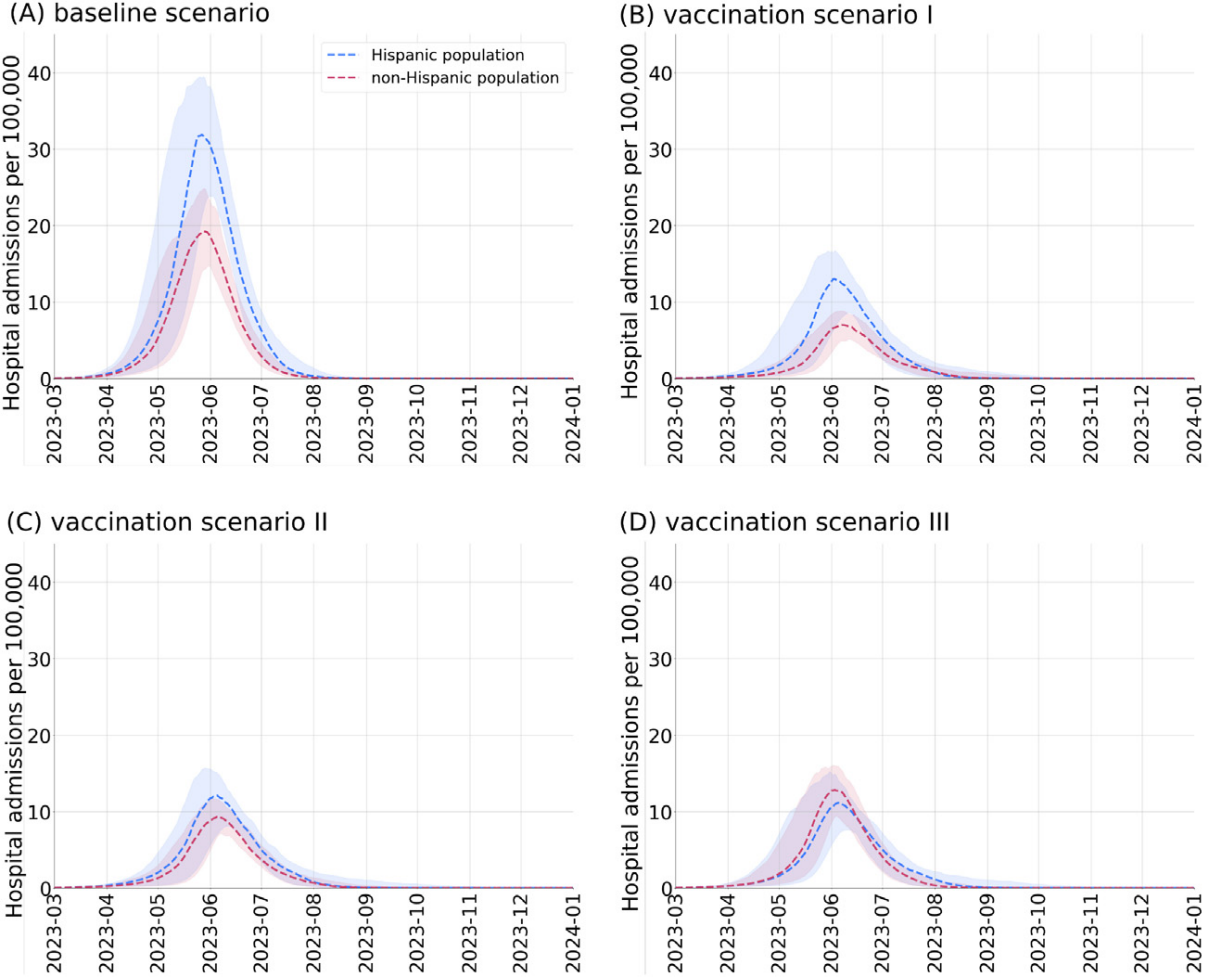
**Projected COVID-19 relative hospitalizations in the Hispanic and non-Hispanic individuals under four scenarios corresponding to vaccination campaigns with bivalent boosters**. The assumed rollout, hesitancy levels and allocation modes for each scenario are provided in Table 2. Ribbons indicate 95% confidence intervals.

**Fig. 5:**
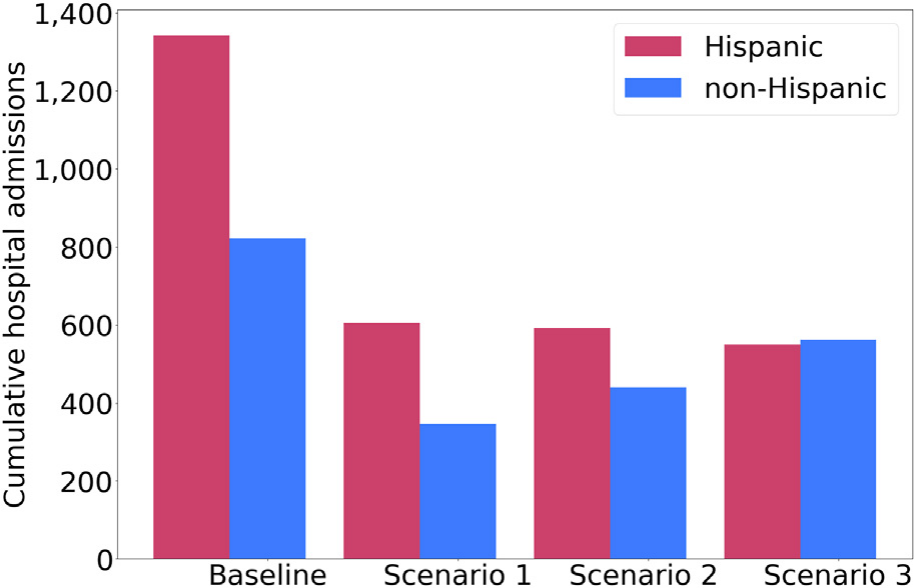
**Bar chart showing the projected cumulative hospital admissions per 100,000 in the Hispanic (pink) and non-Hispanic (blue) populations, under four scenarios of vaccinations**. Cumulative numbers were calculated from Mar 1, 2023, through Dec 31, 2023.

We continue our investigation by looking at the equity of the considered vaccination strategies. We compare the relative number of hospital admissions in the Hispanic and non-Hispanic groups for each strategy. Fig. 4 shows the changes in the relative numbers of hospital admissions in the two groups. Under the baseline vaccination scenario, we expect that the daily hospital admissions among the Hispanic group will be approximately 59% higher than the non-Hispanic one. The equal distribution of vaccines under scenario 1 will further increase the disparities in COVID-19 hospitalizations. We project that the equal allocation of vaccines among Hispanic and non-Hispanic individuals will result in increased relative hospitalization among Hispanic individuals by approximately 88%. While re-allocating half of the doses that are initially assigned the non-Hispanic individuals to Hispanic individuals, under vaccination scenario 2, will reduce the gap in COVID-19 hospitalizations between the two groups to approximately 34%. Finally, reallocating half of the doses that are initially assigned to the non-Hispanic group to the Hispanic individuals will result in slightly reduced relative hospital admissions in this group by approximately 5%. We compared the cumulative hospitalizations in each subgroup under the four considered scenarios in Fig. 5.

## 4 Discussion

This work presents an equity analysis of bivalent booster vaccination strategies that mitigate the emergence of highly transmissible and immune escaping variants in El Paso County, TX. We parameterized a previously developed immuno-epidemiological framework to the recent COVID-19 landscape and stratified it according to the Hispanic and non-Hispanic subpopulations (Bi et al., 2022; Bouchnita et al., 2022a). The advantage of this framework is that it can readily describe the interactions between circulating variants and population immunity in a flexible way. Further, the comparison of the projections made with this framework against retrospective data for hospitalizations and mortality suggests that it provides an accurate estimate for the magnitude of variant-driven surges. We considered that Hispanic individuals are more impacted by COVID-19 because they have higher hospitalization and mortality rates due to the elevated prevalence of COVID-19 risk factors in this group. Further, we captured the elevated risk of infection in the Hispanic population, due to the higher ratio of essential workers in this population using a modified contact matrix. Our first objective was to study the impact of the emergence of highly transmissible and immune escaping variants on the Hispanic population. To achieve this, we considered the emergence of a new variant that has the same characteristics of Omicron BA.1 relative to Delta. Our projections have shown that the relative hospital admissions are approximately 59% higher in the Hispanic group than in their non-Hispanic counterpart. We evaluated the equity of three vaccination strategies corresponding to campaigns that aim to increase the uptake of bivalent boosters but distribute it among Hispanic and non-Hispanic groups according to different modes. We also considered one baseline scenario to evaluate the effectiveness of the vaccination campaigns in mitigating the emergence of the new variant. The three vaccination campaigns effectively mitigated the impact of variant X. Our simulations have shown that the equal allocation of vaccines could increase the existing disparities in COVID-19 hospitalizations. That is because vaccine-derived immunity is more effective at preventing hospitalizations in the less vulnerable group. Prioritizing the Hispanic group in the allocation of bivalent booster vaccines reduces the Hispanic inequities associated with COVID-19. Indeed, reallocating half of the doses initially assigned to the non-Hispanic group to Hispanics effectively reduces the disparity in COVID-19 hospitalizations.

It is important to note that this study is based on a few limitations. First, we restricted the factors driving COVID-19 Hispanic disparities in the relatively higher proportion of COVID-19 comorbidities and essential workers among this group. In reality, there exist other factors contributing to the disproportionate impact of COVID-19 on Hispanic individuals. These factors are increased vaccine hesitancy, reduced access to healthcare, and the higher population density in the geographic areas occupied by Hispanic individuals. We did not consider these three factors because the Hispanic population is predominant in El Paso County, TX. Further, available data show a higher uptake of vaccines in the County, suggesting a high acceptance of vaccines even among Hispanic and non-Hispanic groups. Also, we could not obtain data confirming the high population density in areas occupied by Hispanic individuals in El Paso County, TX. Further, we considered only official estimates provided by the Center for Disease Control regarding the increase in the rate of hospitalization and mortality among Hispanics (CDC, 2023). We make additional scenario projections considering assumptions regarding these differences. Another limitation of the model concerns the effect of the level of immunization and the emergence of new variants on behavior. Our projections do not consider the changes in behavior during the vaccination campaign and before the emergence of highly transmissible variants.

Our results can help policymakers and public health officials in US cities with high Hispanic populations equitably address future surges of COVID-19. The low-dimensionality and scalability of our modeling framework make it an effective tool for assessing the impact of emerging variants, devising mitigation measures tailored to each context, and monitoring the course of the pandemic. Our findings underscore the importance of using vaccinations in a way that protects vulnerable populations and helps reduce COVID-19 disparities.
