# Supplementary material for: Equitable bivalent booster allocation strategies against emerging SARS-CoV-2 variants in US cities with large Hispanic communities: The case of El Paso County, Texas
Source: Infect Dis Model. 2023 Jul 20;8(3):912–9. doi: 10.1016/j.idm.2023.07.009 (PMC10400804; doi:10.1016/j.idm.2023.07.009)
Supplement: Multimedia component 1 [file mmc1.pdf]

Supplementary material

Equitable bivalent booster allocation strategies against emerging SARS-CoV-2 variants in US cities with large Hispanic communities: the case of El Paso County, TX

Francis Owusu Dampare and Anass Bouchnita

Model equations

The model describes the transitions of individuals between several compartments: susceptible (S), exposed (E), pre-symptomatic (PY), pre-asymptomatic (PA), infectious symptomatic (IY), infectious asymptomatic (IA), hospitalized (H), deceased (D), and recovered (R) (Figure 1 in the main document). For each age (*l*), we solve the following equations:

$$\frac{dS_l}{dt} = -S_l \cdot \sum_{i \in A} \frac{\beta_i \phi_{l,i} (I_i^Y + I_i^A \omega^A + P_i^Y + P_i^A \omega^A)}{N_i (1 + \mathbf{K^I}(\mathbf{p}) \mathbf{M^I})} + \eta R$$

$$\frac{dE_l}{dt} = S_l \cdot \sum_{i \in A} \frac{\beta_i \phi_{l,i} (I_i^Y + I_i^A \omega^A + P_i^Y + P_i^A \omega^A)}{N_i (1 + \mathbf{K^I}(\mathbf{p}) \mathbf{M^I})} - \sigma E_l$$

$$\frac{dP_l^A}{dt} = (1 - \tau_l + K_2^1 M_D^I + K_2^2 M_V^I + K_2^3 M_O^I) \sigma E_l - \rho^A P_l^A$$

$$\frac{dP_l^Y}{dt} = (\tau_l - K_2^1 M_D^I - K_2^2 M_V^I - K_2^3 M_O^I) \sigma E_l - \rho^Y P_l^Y$$

$$\frac{dI_l^A}{dt} = \rho^A P_l^A - \gamma^A I_l^A$$

$$\frac{dI_l^Y}{dt} = \rho^Y P_l^Y - (1 - \pi_m) \gamma^Y I_l^Y - \frac{\pi_m \mu I_l^Y}{1 + \mathbf{K^H}(\mathbf{p}) \mathbf{M^H}}$$

$$\frac{dH_l}{dt} = \frac{\pi_m \mu I_l^Y}{1 + \mathbf{K^H}(\mathbf{p}) \mathbf{M^H}} - \gamma_H H_l - \frac{\nu_m H_l}{1 + \mathbf{K^D}(\mathbf{p}) \mathbf{M^H}}$$

$$\frac{dR_l}{dt} = \gamma^A I_l^A + (1 - \pi_m) \gamma^Y I_l^Y + \gamma_H H_l - \eta R_l$$

$$\frac{dD_l}{dt} = \frac{\nu_m H_l}{1+\mathbf{K^D}(\mathbf{p})\mathbf{M^H}},$$

A, represent all possible age groups, ω^A describes the relative infectiousness of the infectious compartments I^A, I^{PA}, β is the transmission rate, $\phi_{a,i}$ is the mixing rate between age group $a,\ i \in A$, and $\gamma^A, \gamma^Y, \gamma^H$ are the recovery rates for the I^A, I^Y, H compartments, respectively, σ is the exposed rate, ρ^A, ρ^Y are the pre-(a)symptomatic rates, τ is the symptomatic ratio, π is the proportion of symptomatic individuals requiring hospitalization, μ is the rate at which hospitalized cases enter the hospital following symptom onset, ν is the mortality rate for hospitalized cases, and η is the rate at which recovered individuals become susceptible again, $\mathbf{K^I(p)}$ = [K^I_{o45}(p), K^I_{oq1}(p), K^I_{oxb}(p), K^I_x(p), K^I_{mb}(p), K^I_{bb}(p)], $\mathbf{K^H(p)}$ = [K^H_{o45}(p), K^H_{oq1}(p), K^H_{oxb}(p), K^H_x(p), K^H_{mb}(p), K^H_{bb}(p)], and $\mathbf{K^D(p)}$ = [K^D_{o45}(p), K^D_{oq1}(p), K^D_{oxb}(p), K^D_x(p), K^D_{mb}(p), K^D_{bb}(p)] are vectors of positive constants that describe the efficacy of immunity in reducing the rates of infection, disease, hospitalization, and death, while $\mathbf{M^I}$ = [M^I_{o45}(p), M^I_{oq1}(p), M^I_{oxb}(p), M^I_x(p), M^I_{mb}(p), M^I_{bb}(p)] and $\mathbf{M^H}$ = [M^I_{o45}(p), M^I_{oq1}(p), M^I_{oxb}(p), M^I_x(p), M^I_{mb}(p), M^I_{bb}(p)] are two vectors consisting of state variables that describe the protection levels derived from vaccination and natural infection against infection and protection, respectively, *p* describes the relative prevalence of each Omicron subvariant.

The model also describes the population-immunity levels generated by natural infections with the variants BA.4/BA.5, BQ.1/BQ.1.1, XBB.1.5, and the hypothetical variant X. For each circulating variant v, we represents the population-immunity gained from infection with variant v which protects against infection and hospitalization as follows:

$$\frac{dM^I_{v,l}}{dt} = k_1 p_v \frac{R_l}{N(1+K_s\mathbf{K^H}(\mathbf{p_v})\mathbf{M^H})} - w^I_1 M^l_{v,l}$$
$$\frac{dM^H_{v,l}}{dt} = k_1 p_v \frac{R_l}{N(1+K_s\mathbf{K^H}(\mathbf{p_v})\mathbf{M^H})} - w^H_1 M^H_{v,l}$$

Where p_v represents the prevalence of the circulating variant v, which could be BA.4/BA.5, BQ.1/BQ.1.1, XBB.1.5, or the hypothetical variant X, w^I_1 and w^H_1 represents the waning rate of immunity gained from infection, and K_s is a saturation constant, which represents the reduction in the generation of antibodies upon infection when the level of immunity is high. Next, we describe the population-immunity generated by monovalent booster vaccination against infection and hospitalization:

$$\frac{dM^I_{mb,l}}{dt} = k_2 B_{mb}(t) - w^I_2 M^l_{mb,l}$$
$$\frac{dM^H_{mb,l}}{dt} = k_2 B_{mb}(t) - w^H_2 M^H_{mb,l}$$

where $B_{mb}(t)$ represent time-dependent functions for the number of administered monovalent boosters, respectively, administered 15 days before, since vaccines require 2 weeks to provide protection, w^I_2 and w^H_2 represents the waning rate of immunity gained from booster vaccination.

we use the same type of equations to describe the population-immunities generated from vaccination with bivalent booster vaccination:

$$\frac{dM_{bb,l}^{I}}{dt} = k_2 B_{bb}(t) - w_2^I M_{bb,l}^{l}$$
$$\frac{dM_{bb,l}^{H}}{dt} = k_2 B_{bb}(t) - w_2^H M_{bb,l}^{H}$$

The model considers that immune escape reduces the efficacy of a type of immunity in reducing susceptibility and severity of another immunity type. Omicron escape to immunity acquired through vaccines and other variants is simulated by reducing the efficacy of immunity against Omicron as follows:

$$K_1^v(p) = K^I(1 - p\epsilon)$$

where *can* be either an Omicron variant or variant X, ϵ represents the levels of Omicron immune escape to infection/symptoms and to severe disease, respectively. We assume that Omicron BA.4/BA.5 do not escape protection against severe disease. The value of ϵ is set such they reduce the rates of infection and symptomatic disease as follows:

$$\frac{r}{1 + K.(1 - p\epsilon)} = (1 - eff)r.$$

We consider that immunity acquired through infection with a specific variant provides the best protection against the same variant [1–3]. Also, we assume that all Omicron variants do not escape immunity acquired by booster shots [4]. The protection levels provided by each time of immunities captured in the model in the absence of immune escape are provided in Table A1.

Table A1. Assumed efficacy levels against the same variant in the absence of immune escape.

Immunity source	Protection against infection	Protection against symptoms	Protection against hospitalization	Protection against death
Infection with Delta	90%	90%	95%	97.5%
Infection with Omicron BA.1, BA.2, BA.12.2.12 and BA.4/BA.5	90%	90%	95%	97.5%
Vaccination with monovalent and bivalent booster shots	88%	88%	95%	97.5%

Parameter values

Numerical values of the epidemiological parameters are provided in Table A2 and values of immunological parameters are presented in Table A3 [5].

Table A2. list of epidemiological parameter values used in the numerical simulations.

Parameters	Value	Source
γ^A: recovery rate on asymptomatic compartment	Equal to γ^Y	Assumption
γ^Y: recovery rate on symptomatic non-treated compartment	0.25	[6]
τ: symptomatic proportion (%)	0.35	Adjusted to have 1 symptomatic case out of 4 in the steady-state for Delta
σ: exposed rate	1/1.5	increased from 1/2.9 to 1/1.5 because of Delta [3]
ρ^A: pre-asymptomatic rate	Equal to ρ^Y	
ρ^Y: pre-symptomatic rate	$\frac{1}{2.3}$	[6]
ω^A: relative infectiousness of infectious individuals in compartment I^A	$\frac{2}{3}$	[7]
IFR: non-Hispanic infected fatality ratio, age specific (%)	[0.0009, 0.0022, 0.0022, 0.0339, 0.2520, 0.6440]	Age adjusted from Verity et al. [8]
YFR: non-Hispanic symptomatic fatality ratio, age specific (%)	[0.001608, 0.003823, 0.003823, 0.05943, 0.4420, 1.130]	$YFR = \frac{IFR}{\tau}$

Table A2. list of immunological parameter values used in the numerical simulations.

Rate of population immunization from natural infections (k_1)	153.55	Fitted to multiscale model results
Rate of population immunization from vaccination (k_2)	0.112	Fitted to data
Constant of saturation from natural infection (K_s)	100	Fitted to multiscale model results

Age-specific contact patterns

Contact matrices for the US are used to describe mixing patterns between age groups [9]. The model uses three matrices to describe the contact patterns in all locations, schools and workplaces in order to represent the reduction in mobility during holidays and weekends. We consider that schools close during weekends and from December 18 to January 02 and also during the months of June, July and August. Workplaces are considered to be closed during the weekends. Hispanic individuals have more essential workers and make more contacts at work. We calculate their specific contact matrix at work by assuming a higher prevalence of frontline workers in the Hispanic community, and that essential workers make 4.5 more contacts per day than non-essential workers.The overall contact matrix for non-Hispanic and Hispanic individuals are calculated as follows:

$$CM^{nH} = CM_{all}^{nH} - \alpha_s(t)CM_s - \alpha_w(t)CM_w^{nH},$$

$$CM^{H} = CM_{all}^{H} - \alpha_s(t)CM_s - \alpha_w(t)CM_w^{H},$$

where $CM^{nH}{}_{all}$,$CM^{H}{}_{all}$ are the contact matrices in all locations for Hispanic and non-Hispanic individuals, respectively. $CM^{nH}{}_{w}$,$CM^{H}{}_{wl}$ are the contact matrices in workplaces for Hispanic and non-Hispanic individuals, respectively, and CM_s represents the contact matrix in schools, taken to be similar for Hispanic and non-Hispanic individuals. $\alpha_s(t)$ *and* $\alpha_w(t)$ are time-dependent functions that describe the opening or closure of schools and workplaces, they take the value of 0 if the corresponding location is opened and 1 if it is closed. The considered contact matrices are as follows:

$$CM_{all}^{nH} = \begin{bmatrix} 2.598237 & 1.600682 & 0.1895988 & 4.1198752 & 0.912514 & 0.112739 \\ 0.640235268 & 8.428533343 & 0.400015072 & 4.028603965 & 0.709643468 & 0.103204179 \\ 0.173684 & 2.0999574 & 6.663684 & 8.710766 & 0.5601588 & 0.0327582 \\ 0.490443671 & 1.516968944 & 0.759891199 & 10.27014274 & 1.714438659 & 0.095919246 \\ 0.431143971 & 1.339346998 & 0.592373724 & 6.379632659 & 3.196133287 & 0.188612431 \\ 0.204998347 & 0.718001781 & 0.182731115 & 2.136319698 & 1.558267141 & 0.602532372 \end{bmatrix},$$

$$CM_{all}^{H} = \begin{bmatrix} 2.598237 & 1.600682 & 0.1895988 & 4.1198752 & 0.912514 & 0.274027 \\ 0.64023527 & 8.43272098 & 0.40062327 & 4.03828082 & 0.71028995 & 0.10320445 \\ 0.173684 & 2.10208136 & 6.70437733 & 8.88619638 & 0.57454777 & 0.03275838 \\ 0.49044367 & 1.52296103 & 0.77794698 & 10.78548093 & 1.79788433 & 0.09592088 \\ 0.43114397 & 1.34667791 & 0.59994777 & 6.64565372 & 3.27076049 & 0.18861448 \\ 0.20499857 & 0.71800217 & 0.1827319 & 2.13634804 & 1.5582765 & 0.60253451 \end{bmatrix},$$

$$CM_s = \begin{bmatrix} 1.196597632 & 0.269627261 & 0.03173379 & 0.38262616 & 0.049755762 & 0 \\ 0.139739606 & 3.973684579 & 0.051319078 & 0.369792419 & 0.075075384 & 0.000263253 \\ 0.016961126 & 0.903246574 & 3.427856164 & 2.582830513 & 0.060321191 & 0 \\ 0.058180033 & 0.331477088 & 0.188215674 & 0.461408137 & 0.042344186 & 0.000352703 \\ 0.093904827 & 0.568170143 & 0.243358213 & 0.35953993 & 0.073783363 & 0.0005338 \\ 0.000729122 & 0.021954765 & 0.006167126 & 0.029787663 & 0.03474166 & 0.011651215 \end{bmatrix},$$

$$CM_w^{nH} = \begin{bmatrix} 0 & 0 & 0 & 0 & 0 & 1.20585 \times 10^{-05} \\ 0 & 0.039768604 & 0.005775822 & 0.091897952 & 0.006139445 & 0 \\ 0 & 0.020170591 & 0.386451333 & 1.666005478 & 0.136647372 & 0 \\ 0 & 0.056904943 & 0.171469933 & 4.893999929 & 0.792456512 & 0 \\ 0 & 0.069619305 & 0.071928236 & 2.526315884 & 0.70871039 & 0 \\ 0 & 0 & 0 & 0.00026916 & 8.88673 \times 10^{-05} & 2.02847 \times 10^{-05} \end{bmatrix}.$$

$$CM_w^{H} = \begin{bmatrix} 0 & 0 & 0 & 0 & 0 & 1.33282 \times 10^{-05} \\ 0 & 0.043956 & 0.0063840 & 0.101574 & 0.0067859 & 0 \\ 0 & 0.022294 & 0.42714 & 1.84143 & 0.1.51036 & 0 \\ 0 & 0.062897 & 0.189525 & 54093 & 0.87590 & 0 \\ 0 & 0.0769502 & 0.079502 & 2.7923 & 0.783337 & 0 \\ 0 & 0 & 0 & 0.0002975 & 9.8225 \times 10^{-05} & 2.2420 \times 10^{-05} \end{bmatrix}.$$

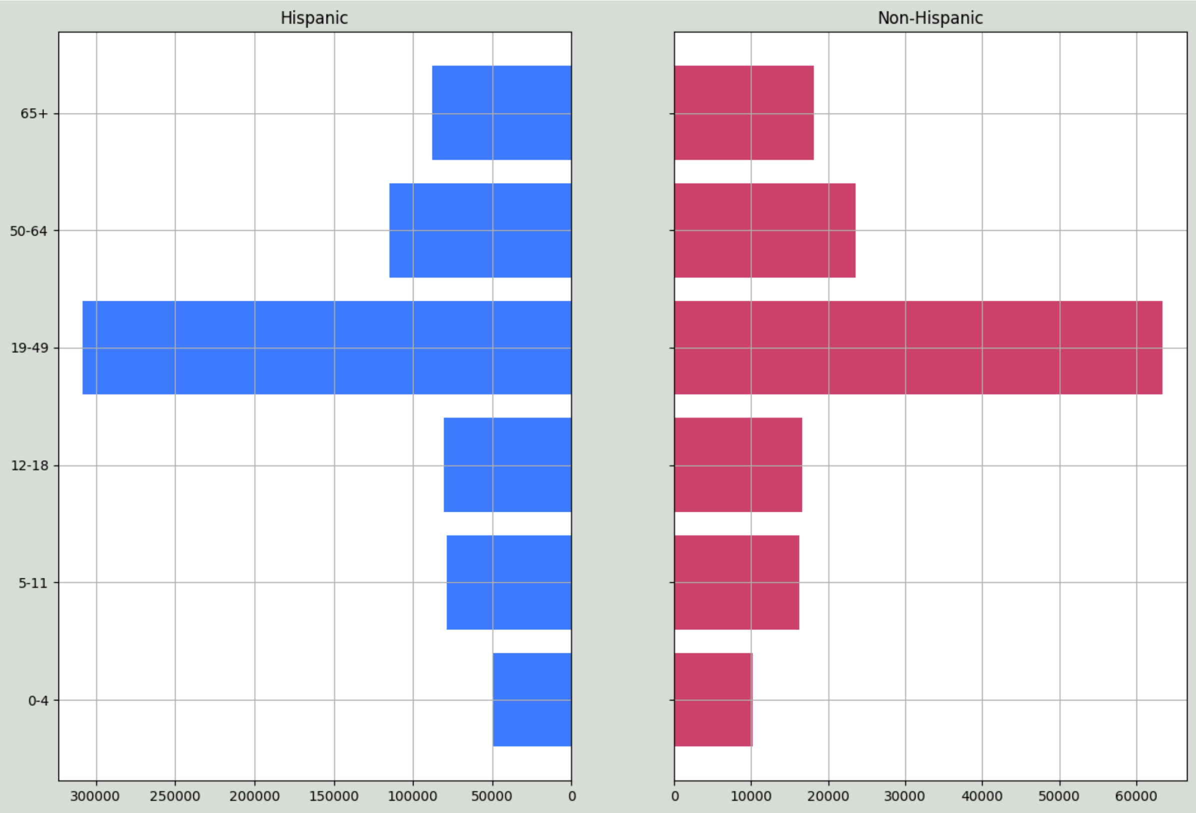

Figure S1: The age tree of the Hispanic and non-Hispanic subpopulations considered in the model.

Stochastic projections

Microstochasticities are introduced using the Euler-Maruyama Method. Furthermore, the daily transmission rate is sampled from the distribution $N(\beta_F, \sigma_\beta)$, where β_F is the transmission rate for

the fitting period, σ_β describes the difference between the 95% confidence interval and the median for the fitted transmission rates values during the fitting period.

For each scenario projection, we made 100 simulation runs and computed the 7-day rolling averages. Then, the 0.05, 0.50, 0.95 quantities are computed for each day.

Demographic data

Using Census data, we estimated the population of El Paso County, TX at 867,979. 82.93% of the population is Hispanic. Most of the individuals living in El Paso County, TX belong to the 19-49 age group. We present the considered number of individuals belonging to each age group in the Hispanic and non-Hispanic subpopulations in Figure S1.

References

[1] S. Gazit, R. Shlezinger, G. Perez, R. Lotan, A. Peretz, A. Ben-Tov, D. Cohen, K. Muhsen, G. Chodick, T. Patalon, (n.d.).

[2] M. Šmíd, L. Berec, O. Májek, T. Pavlík, J. Jarkovský, J. Weiner, L. Přibylová, T. Barusová, J. Trnka, (n.d.).

[3] K. Stiasny, I. Medits, D. Springer, M. Graninger, J. Camp, E. Höltl, S. Aberle, M. Traugott, W. Hoepler, J. Deutsch, O. Lammel, C. Borsodi, A. Zoufaly, L. Weseslindtner, J. Aberle, E. Puchhammer-St , (n.d.).

[4] L.J. Abu-Raddad, H. Chemaitelly, H.H. Ayoub, S. AlMukdad, H.M. Yassine, H.A. Al-Khatib, M.K. Smatti, P. Tang, M.R. Hasan, P. Coyle, Z. Al-Kanaani, E. Al-Kuwari, A. Jeremijenko, A.H. Kaleeckal, A.N. Latif, R.M. Shaik, H.F. Abdul-Rahim, G.K. Nasrallah, M.G. Al-Kuwari, A.A. Butt, H.E. Al-Romaihi, M.H. Al-Thani, A. Al-Khal, R. Bertollini, N. Engl. J. Med. 386 (2022) 1804–1816.

[5] M. Gilbert, Oxford Music Online (2003).

[6] X. He, E.H.Y. Lau, P. Wu, X. Deng, J. Wang, X. Hao, Y.C. Lau, J.Y. Wong, Y. Guan, X. Tan, X. Mo, Y. Chen, B. Liao, W. Chen, F. Hu, Q. Zhang, M. Zhong, Y. Wu, L. Zhao, F. Zhang, B.J. Cowling, F. Li, G.M. Leung, Nat. Med. (2020).

[7] D. He, S. Zhao, Q. Lin, Z. Zhuang, P. Cao, M.H. Wang, L. Yang, Int. J. Infect. Dis. 94 (2020) 145–147.

[8] R. Verity, L.C. Okell, I. Dorigatti, P. Winskill, C. Whittaker, N. Imai, G. Cuomo-Dannenburg, H. Thompson, P. Walker, H. Fu, A. Dighe, J. Griffin, A. Cori, M. Baguelin, S. Bhatia, A. Boonyasiri, Z.M. Cucunuba, R. Fitzjohn, K.A.M. Gaythorpe, W. Green, A. Hamlet, W. Hinsley, D. Laydon, G. Nedjati-Gilani, S. Riley, S. van-Elsand, E. Volz, H. Wang, Y. Wang, X. Xi, C. Donnelly, A. Ghani, N. Ferguson, Epidemiology (2020).

[9] K. Prem, A.R. Cook, M. Jit, PLOS Computational Biology 13 (2017) e1005697.
